# A theoretical quantitative model for evolution of cancer chemotherapy resistance

**DOI:** 10.1186/1745-6150-5-25

**Published:** 2010-04-20

**Authors:** Ariosto S Silva, Robert A Gatenby

**Affiliations:** 1 Department of Radiology and Integrative Mathematical Oncology, Moffitt Cancer Center, Tampa, FL, USA

## Abstract

**Background:**

Disseminated cancer remains a nearly uniformly fatal disease. While a number of effective chemotherapies are available, tumors inevitably evolve resistance to these drugs ultimately resulting in treatment failure and cancer progression. Causes for chemotherapy failure in cancer treatment reside in multiple levels: poor vascularization, hypoxia, intratumoral high interstitial fluid pressure, and phenotypic resistance to drug-induced toxicity through upregulated xenobiotic metabolism or DNA repair mechanisms and silencing of apoptotic pathways. We propose that in order to understand the evolutionary dynamics that allow tumors to develop chemoresistance, a comprehensive quantitative model must be used to describe the interactions of cell resistance mechanisms and tumor microenvironment during chemotherapy.

Ultimately, the purpose of this model is to identify the best strategies to treat different types of tumor (tumor microenvironment, genetic/phenotypic tumor heterogeneity, tumor growth rate, etc.). We predict that the most promising strategies are those that are both cytotoxic and apply a selective pressure for a phenotype that is less fit than that of the original cancer population. This strategy, known as double bind, is different from the selection process imposed by standard chemotherapy, which tends to produce a resistant population that simply upregulates xenobiotic metabolism. In order to achieve this goal we propose to simulate different tumor progression and therapy strategies (chemotherapy and glucose restriction) targeting stabilization of tumor size and minimization of chemoresistance.

**Results:**

This work confirms the prediction of previous mathematical models and simulations that suggested that administration of chemotherapy with the goal of tumor stabilization instead of eradication would yield better results (longer subject survival) than the use of maximum tolerated doses. Our simulations also indicate that the simultaneous administration of chemotherapy and 2-deoxy-glucose does not optimize treatment outcome because when simultaneously administered these drugs are antagonists. The best results were obtained when 2-deoxy-glucose was followed by chemotherapy in two separate doses.

**Conclusions:**

These results suggest that the maximum potential of a combined therapy may depend on how each of the drugs modifies the evolutionary landscape and that a rational use of these properties may prevent or at least delay relapse.

**Reviewers:**

This article was reviewed by Dr Marek Kimmel and Dr Mark Little.

## Background

Disseminated cancer remains a nearly uniformly fatal disease. While a number of initially effective chemotherapies are available, tumors inevitably develop resistance to these drugs ultimately resulting in treatment failure and cancer progression. Causes for chemotherapy failure in cancer treatment reside in multiple levels: poor vascularization, hypoxia, intratumoral high interstitial fluid pressure, and phenotypic resistance to drug-induced toxicity through up regulated xenobiotic metabolism or DNA repair mechanisms and silencing of apoptotic pathways [1-5].

Solid tumors may present both phenotypic and environmental therapy resistance. Phenotypic resistance is due to increased cell survival mechanisms, environmental resistance consists in reduced drug efficiency by tumor microenvironmental conditions. Examples of environmental resistance in solid tumors are hypoxia -which reduces efficiency of radiotherapy-, slow diffusion of drugs from blood into avascular regions of tumors and pHe induced quiescence [6].

Clinical tumors are rarely detected before they reach a size of 1 cubic centimeter so that even a minimum tumor burden will contain around 109 cells [7]. In view of the intrinsic genetic instability that is characteristically observed in cancer phenotypes, a billion cells will form a phenotypically and genotypically heterogeneous population which may harbor small populations of cells which are already chemoresistant. In other words, phenotypes with at least some degree of resistance to therapy are likely to be present even prior to its administration.

Frequently, the initial doses of chemotherapy eradicate a significant fraction of the tumor population. However, most tumors typically become resistant over time resulting in repopulation of the original tumor site and development of other metastases [8].

Unless a cytotoxic therapy eradicates all cancer cells, its application to a tumor population also produces evolutionary selection forces that will select for the individuals that are adapted to the therapy and, thus, fittest to these conditions. In fact, this mechanism has been used to produce many chemoresistant cells lines [9-11].

A fundamental principle of chemotherapy is to use drugs that are more toxic to tumor cells than to healthy cells, the preferred target being replication mechanisms, as many tumors replicate faster than the host tissue (except for fast replicating tissue such as epithelium). Unfortunately, tumors are not homogenously proliferative. Typically, only its outer rim is composed of replicating cells, while much of its mass consists of cells in quiescent or even dying states [12].

Thus, the cells on the outer rim of the tumor are the fittest extant phenotype in the tumor in absence of treatment. They are also the most readily targeted by chemotherapy due to their proximity to vascularization and their fast growth. This region of the tumor is thus environmentally sensitive and is mostly composed of phenotypically sensitive cells, even though some phenotypically resistant cells may be present.

The inner regions of a solid avascular tumor are often hypoxic and acidic due to anaerobic glucose metabolism, what leads to quiescence and increased chemoresistance. The increasing distance from vascularization reduces the concentration of drug in these regions of tumor, conferring a second factor of environmental resistance [13,14].

It has been proposed that chemoresistant cells are typically, in the absence of therapy, less fit that chemosensitive cells. This is due to the phenotypic cost of resistance. That is, the resistance to cytotoxicity requires expenditure of resources and energy to upregulate xenobiotic metabolism or DNA repair pathways. This energy is, thus, not available for proliferation resulting in a reduction in the fitness. Assuming that phenotypically resistant cells are less fit and, therefore, less proliferative than their chemo sensitive counterparts [15,16] -in this model we consider chemotherapy targeting mainly proliferative cells- it is reasonable to consider that most of phenotypically chemoresistant cells will be trapped inside the tumor volume as the latter grows.

Consequently we propose that the inner region of a tumor confers environmental resistance to cells there residing due to reduced blood flow, hypoxia, and acidosis and that it also harbors most of the phenotypically resistant cells as well, making this area a candidate for origin of tumor regrowth and source of chemo resistance.

### Evolutionary double bind and cancer therapy

In this work we describe and model solid tumors as a phenotypically heterogeneous cell population distributed in an environmentally heterogeneous microenvironment which supports proliferation when possible but also rewards quiescence when needed.

Such a plastic evolutionary landscape shows similarities to the control of invasive pests with chemicals: at first, chemicals are capable of eradicating the pests almost entirely, but after some generations, an entire population of resistant individuals arises.

Centuries of experience in dealing with damaging exotic species have demonstrated that best strategy is to introduce into the ecosystem a predator, pathogen, or parasitoid that targets the foreign population but not the native species. These strategies work best when the invasive species can adapt to the biological attack through complex and costly phenotypic changes that substantially reduce its fitness. This state, in which the pest faces either attack by an enemy or costly adaptation that reduces its ability to proliferate, has been termed an evolutionary double bind [17].

The idea of changing cancer from a 'must cure' disease to a chronic disease has been recently proposed by this group [18] through computer and mathematical models and tested in animal models showing promising results. One potentially valuable strategy to achieve this involves reducing the fitness of the tumor without actually causing cytotoxicity. This could potentially stabilize the cancer population without producing strong selective forces for resistant phenotypes.

One specific form of this therapeutic strategy might take advantage of the glycolytic metabolism commonly observed in human cancers. Studies reporting the use of glucose competitors such as 2-deoxyglucose to treat solid tumors have been published for close to three decades [19,20] and rely on the knowledge that the cells in tumor center regions are highly dependent on anaerobic glucose metabolism due to hypoxia reigning in this region.

The rationale behind this strategy is that the same hypoxic and acidic environment that confers environmental chemoresistance to these cells also forces them to increase their glycolytic metabolism to compensate for the loss of energy due to reduced respiration described as Pasteur Effect when this modification is transient and Warburg Effect when it becomes independent of oxygen availability [21]. These cells are more sensitive to glucose restriction than healthy tissue or tumor cells closer to tumor-host interface.

We propose that the increased robustness to chemotherapy provided by tumor hypoxic core also confers fragility against glucose restriction and that this trade-off can be instrumental to a double-bind strategy for treatment.

### Double bind applied to solid tumor treatment

In this work we propose to approach solid tumors chemotherapy using an evolutionary double bind strategy that weakens cells in both tumor rim and center, forcing sensitive and resistant cells to compete with each other leading to tumor stabilization, in worst case, or eradication, in best case scenario.

This approach assumes that the resistant phenotypes are rare in population [22] and that resistance has a cost leading to slower proliferation in free growth conditions what eventually leads to phenotypically resistant cells trapped in the inner regions of tumor while sensitive proliferative cells are located in outer rim.

The strategy adopted in our model consists in targeting the resistant cells with glucose competitors and lead them to energy depletion and consequent cell death. The tumor is kept stable with low doses (1 μM*h corresponding to IC_99 _of sensitive cells or IC_50 _of resistant subpopulation) in order to prolong patient survival until the resistant population has been eradicated by competition with sensitive ones.

We also evaluate the efficiency of different combinations of chemotherapy and glucose competitor and the effect of half-life of these drugs in system.

This is the first work, to our knowledge, that proposes to identify the ideal conditions of combination of glucose restriction and chemotherapy together with an analysis of the progress of tumor resistance (phenotypically and environmentally).

## Methods

The model used in this work consists of a bidimensional fixed-lattice cellular automata representation of a tumor mass which was allowed to grow until it reached the size of approximately 5,000 cells before treatment was started (each cell represented as a 25 μm-side square), corresponding to a total tumor diameter of 2 mm.

Each cell in the tumor had its intrinsic phenotypic resistance and proliferative capacity which, according to this model's assumption, are inversely correlated. In this study, the following scenario was tested: sensitive population with proliferative potential of 1 and IC_50 _of 10 nM*h and resistant population with proliferative potential of 0.05 and IC_50 _of 1 μM*h.

A proliferative potential equal to 1 means that at every generation, if a cell has enough energy and space to replicate, it will do so. If a cell has a proliferative potential of 0.5, it can only replicate as fast as once every two generations of simulation and a cell with 0.05 will proliferate once every 20 generations.

In this model, the interval between two treatment sessions was arbitrarily defined as 5 generations, which in human tumor cells would correspond to 1 month.

### Diffusion, cell cycle, tumor growth

The proposed tumor is avascular and its growth is limited by its cell's proliferative rate and energy availability, which is a function of glucose and oxygen in interstitial fluid.

Oxygen, glucose and pH buffers diffuse through the entire simulated volume isotropically and their concentrations are considered constant in blood vessels which are the boundary conditions of this model [23]. The concentration of species in blood serum and diffusion constants are listed in table 1[24-29].

**Table 1:** Constants and values used in model

Constant	Value	Reference
H^+ ^diffusion coefficient	1.08 × 10^-5 ^cm^2^/s	[24]
O_2 _diffusion coefficient	1.46 × 10^-5 ^cm^2^/s	[25]
Glucose diffusion coefficient	5 × 10^-6 ^cm^2^/s	[26]
O_2 _uptake rate	9.41 × 10^-2 ^× [O_2_]/s	[27]
Glucose uptake rate	5 × 10^-5 ^× [Glc]/s	[24]
Quiescence pH threshold for normal cells	7.1	[28]
Death pH threshold for normal cells	6.8	[28]
Mutation rate for tumor cells	0.1%	[28]
ATP production for 100% probability of cell duplication.	8.6 × 10^-6 ^M/s	[29]
Minimum ATP production for cell survival.	8.6 × 10^-7 ^M/s	[29]

In order to represent the gradient of nutrients from vascularized tissue to tumor we represented the external surface around tumor as vascularized tissue. This tissue does not have fixed values for glucose, oxygen and other species as blood vessels.

As tumor grows, it replaces this vascularized tissue. Once therapy is applied to the system, the tumor recedes but it loses contact with the vascularized tissue, until it re-grows and gets in contact with it again.

The drug levels in blood were simplified to a step function where the concentration of drug is kept constant during treatment and then the drug is completely removed from system. The real behavior of drug concentration in blood is an initial spike followed by an exponential decay [30,31] which we approach by the equation 1:

Where T_1/2 _is the half life of drug in blood and t is time. Integrating this function to infinity we obtain the AUC (Area under curve of concentration versus time). So, the width of a step function with the same AUC and bolus intensity is given by:

In this work, simulations of treatment considered a duration of 5,000 s for drug exposure step function, corresponding to an AUC with exponential decay with half life of approximately one hour, which is close to drugs such as carboplatin (1-2 h).

### Chemoresistance

Chemotherapy was represented as a hypothetical drug with diffusion coefficient similar to glucose a half-life in the order of one hour and having fast replicating cells as target.

Drug resistance was implemented as a generalization of the definition of IC_50_, which represents the drug concentration and exposition time for which half the original population survives. In this model we consider that the probability of one specific cell to survive is given by the dose response curve, meaning that if the extracellular drug concentration for a given cell is IC_50 _the cell has a chance of 50% of survival.

Each simulation step could be either (a) tumor free growth or (b) therapy. During tumor free growth, no drug is present and tumor cells are allowed to proliferate restricted only by its energetic capacity, pH induced quiescence, proliferation rate and free space to grow.

During therapy, however, cells are subject to death induced by chemotherapy as previously described, suffer competition of its glucose transporters by 2-deoxy-glucose and thus have their energetic capacity reduced potentially leading to death by energy depletion or may suffer low pHe induced death.

### Metabolism, replication and cell death

In this model cell metabolism consisted in converting glucose and oxygen into ATP, carbon dioxide and lactic acid. Glucose and oxygen uptakes were proportional to extracellular concentration [28].

In normoxic conditions, each molecule of glucose combined with six molecules of oxygen to produce 36 molecules of ATP and 6 molecules of CO_2_. In hypoxic conditions the excess of glucose was metabolized anaerobically producing two molecules of ATP and two of lactic acid.

Lactic acid (pK_a _= 3.86) was considered to dissociate completely under physiological pH (~7.4). The extracellular pH (pH_e_) in blood was fixed at 7.2 as was serum glucose (5 mM) and oxygen (0.15 mM corresponding to 100 mmHg pO_2_) concentrations.

Low pH_e _could induce cell quiescence (pH_e _< pH_Q_, pH quiescence threshold) or cell death (pH_e _< pH_D_, pH death threshold) [32-34]. The values of pH_Q _and pH_D _are randomly distributed among the cell population according to a uniform distribution within the following intervals: 6.0 < pH_D _< 6.8 and 6.4 < pH_Q _< 7.1.

Cell proliferation rate was connected to its metabolic capacity [29]. If ATP production was below a minimum threshold (0.85 uM/s), the cell would die. Otherwise, if ATP production was above this minimum threshold the cell would have an increasing probability of replication, reaching 100% at a maximum threshold (8.5 uM/s).

As previously described, cells with higher proliferative phenotype have shorter cell cycle but will only replicate if the energy requirements are met.

### Simulations

In all simulations, the model was created initially with cells with two subpopulations: one resistant and with low proliferation rate and a second chemosensitive and highly proliferative.

The tumor disc was allowed to grow until 5,000 cells (corresponding to 270,000 cells in a spherical volume) before the following scenarios were tested: (a) no therapy, (b) high dose chemotherapy aiming tumor eradication, (c) 2-deoxy-glucose only therapy, (d) simultaneous administration of low dose chemotherapy with 2-deoxy-glucose and (e) alternated administrations of chemotherapy and glucose competitor.

### Double Bind and MTD

High dose chemotherapy (maximum tolerated dose, MTD) concentration was 5-fold the IC_50 _of the chemoresistant population (7 μM*h). And was allowed to permeate the system for 5,000 s (1 h23 m). This number of simulation steps was chosen arbitrarily in order to provide enough time for drug to diffuse to tumor center but also short enough to comply with the half life of known drugs.

**Figure 1:**
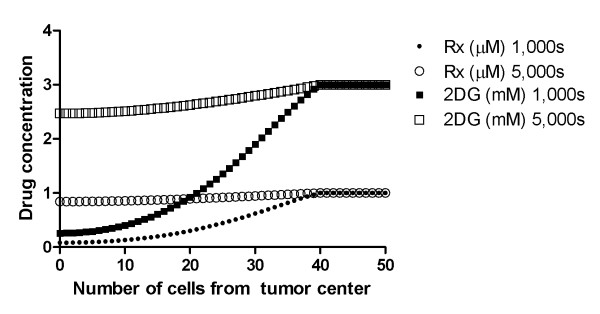
**Gradient of drug concentration for both chemotherapy and glucose competitor**. Gradient of drug concentration for both chemotherapy and glucose competitor. The longer the system is exposed to drugs, the higher the concentration in tumor center and more efficient the treatment.

This combination of diffusion coefficient and drug exposure time leads to the gradients in figure 1, where the concentration of drug in center of tumor is 80% of the one in blood.

For the Double Bind therapy, the minimum amount of drug was used to keep tumor size stable. The dose applied was 20% of MTD, or 1.4 μM*h. After administering the dose, tumor was allowed to re-grow and higher doses were used if tumor size was bigger than original dimensions or lower doses if the tumor was smaller then the original. If no growth was detected no therapy was administered.

### 2-Deoxy-Glucose

2-deoxy-glucose therapy was tested for concentrations in the order of magnitude of glucose in blood serum. The maximum dose, in the model, was determined to be 3 mM. Higher concentrations induced death of cells in healthy tissue further than 150 μm from blood vessels due to energy depletion. In animal experiments chronic doses (diet) of 150 μM/l and bolus (i.p. injection) of 12 mM [35,36] have been used.

In all simulations in this work, 2-deoxy-glucose was allowed to diffuse in model for 5,000 s and the concentration in center of tumor was around 85% of the concentration in tumor rim (2.5 mM and 3 mM respectively).

### Combination of strategies

We also tested if the alternative combinations of chemotherapy and 2-deoxy-glucose would have better results than the simultaneous administration. The combination was tested as (a) chemotherapy and 2-deoxy-glucose administered in tandem, (b) 2-deoxy-glucose administration followed by chemotherapy and (c) chemotherapy followed by 2-deoxy-glucose.

## Results

Previous in vitro and in vivo works [36] have shown that exposing tumor cells to 2-deoxy-glucose concentrations on the order of those considered in this study combined with chemotherapy could provide better results than chemotherapy alone. These studies, however, used simultaneous administration of these drugs and did not consider sequential application.

Recent theoretical/in vitro models [37] have pointed the diffusion of chemotherapeutics through tumor towards its center as one of the limitations of success in solid tumor treatment. According this model the concentration of drug can decrease 50% at a distance of three cells from tumor-host interface in steady-state and up to 80% across the same distance in a transient of 2 hours.

The gradients of drug concentration in our models reached steady state after 5,000 s (~1.5 h) and corresponded to a decrease in concentration of the order of 15% across 40 cells.

Our simulations have showed that administration of 2-deoxy-glucose at 3 mM reduces ATP production levels in tumor cells in outer rim from 7.4 μM/s to 3.8 μM/s and hypoxic core from 2.7 μM/s to 0.9 μM/s and a loss of approximately 3,500 cells due to energy depletion (ATP production levels lower than 8.5 μM/s).

Previous experiments [36] have shown in vitro that the use of 3 mM 2-deoxy-glucose decreases ATP levels in the order of 60% in normoxic conditions and 70% in hypoxic conditions, our simulations showed a decrease in 50% under normoxic conditions and 70% in hypoxic, showing agreement with in vitro experimental data.

### Control tumor

**Figure 2:**
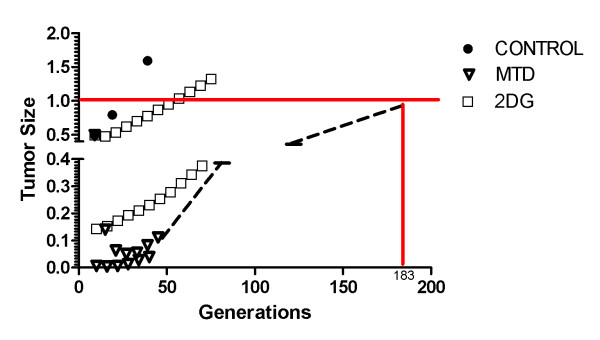
**Tumor growth for untreated (CONTROL), maximum tolerated dose treatment (MTD) and 2-deoxy-glucose only treatment (2DG)**. Tumor growth for untreated (CONTROL), maximum tolerated dose treatment (MTD) and 2-deoxy-glucose only treatment (2DG). Sizes are normalized to 1 being maximum tumor burden. Untreated tumors reach limit size after 26 generations; 2DG reaches this size after 57 generations and MTD after 183 generations.

**Figure 3:**
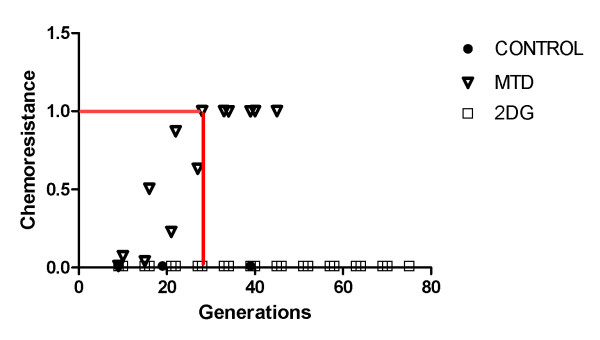
**Average tumor chemoresistance for untreated (CONTROL), maximum tolerated dose treatment (MTD) and 2-deoxy-glucose only treatment (2DG)**. Average tumor chemoresistance for untreated (CONTROL), maximum tolerated dose treatment (MTD) and 2-deoxy-glucose only treatment (2DG). Only tumor treated with chemotherapy will "develop" chemoresistance to treatment as the treatment will change adaptive landscape of tumor to favor cells with resistant phenotype. In fact, the untreated tumor also contains resistant cells but these are kept in small numbers in absence of therapy.

Without treatment, the tumor reached the lethal size (10,000 cells in simulated disc corresponding to 700,000 in spherical volume) in 26 generations, equivalent to 6 months. During free growth, fast proliferating chemosensitive cells are selected and thus the average chemoresistance of the tumor decreases gradually while proliferative phenotype value increases (figures 2 and 3).

**Figure 4:**
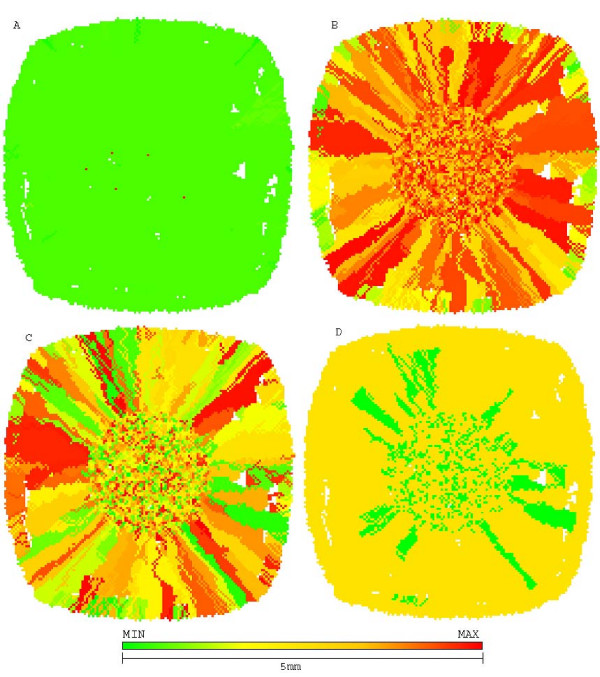
**Growth pattern of untreated tumor at generation 39**. Growth pattern of untreated tumor at generation 39. A shows chemosensitive cells (green) and chemoresistant cells (red). B shows distribution of glycolytic phenotype: cells closer to green have slower metabolic rate and those closer to red are more glucose avid. C shows distribution of acid resistance, the closer to red the more resistant and the ones closer to green are more sensitive to low pHe. D shows cells in low pHe induced quiescent state in green and proliferative cells in yellow.

Figure 4 shows the morphology of this untreated tumor, with a few chemoresistant cells in center while most of tumor mass is composed of chemosensitive cells. The sensitive population, however, is composed of other subpopulations with different phenotypes for glucose metabolism and acid resistance, some proliferating while others are quiescent.

### MTD

The use of high dose chemotherapy, equivalent to 5 times IC_50 _of chemoresistant population and 500-fold higher than IC_50 _of chemosensitive cells leads to death of chemosensitive cells and consequent tumor size reduction (figure 2), but also an increase in the average tumor chemoresistance due to increased population share of chemoresistant cells (figure 3).

**Figure 5:**
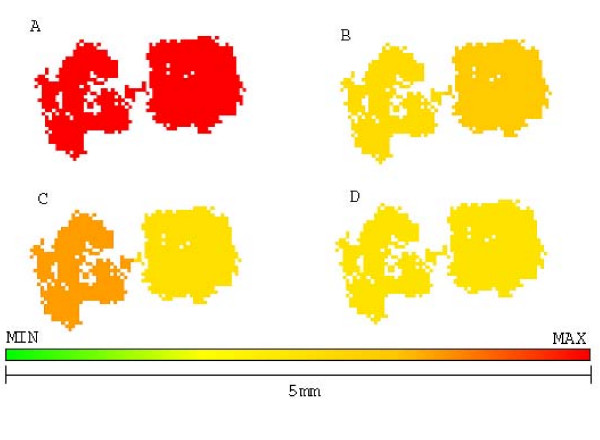
**Growth pattern of a tumor treated with maximum tolerated dose at generation 45**. Growth pattern of a tumor treated with maximum tolerated dose at generation 45. A represents chemoresistance (red resistant and green sensitive), B represents hyperglycolysis (red high glycolysis and green low), C shows acid resistance (red resistant to low pHe and green sensitive) and D shows quiescent cells (green) and proliferative (yellow). Despite being 10 times smaller than the original tumor, it is entirely composed of chemoresistant cells (red) and will not respond to therapy.

The repeated use of this strategy eventually creates a chemoresistant tumor (figure 5) that reaches lethal size in 183 generations (approximately 3.5 years), with low response to therapy.

### 2DG only

**Figure 6:**
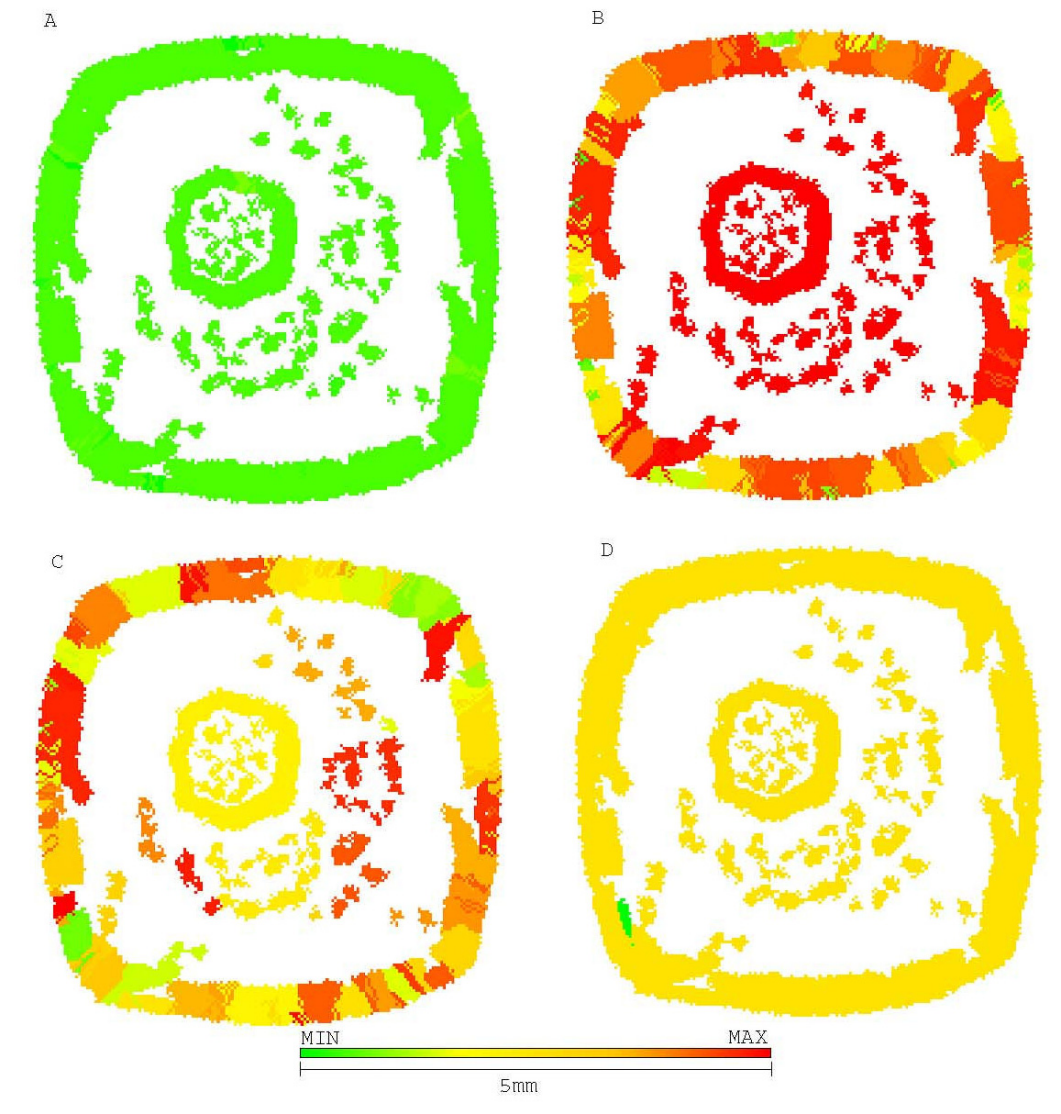
**Growth pattern of 2DG treated tumors at generation 57**. Growth pattern of 2DG treated tumors at generation 57. A represents chemoresistance (red resistant and green sensitive), B hyperglycolysis (red high glycolysis and green low), C shows acid resistance (red high resistance to low pHe and green sensitivity) and D shows quiescent (green) and proliferative (yellow) cells. Treatment with 2DG may lead to eradication of chemoresistant cells but does not prevent tumor growth.

2-deoxy-glucose administered alone was capable of reducing the number of cells in tumor short after administration (5,000 cells to 1,500) without increasing chemoresistance. However, all dead cells were located in tumor interior (figure 6), and thus were not in proliferative state due to lack of free space to grow into. The external tumor volume, thus, and its growth rate were only moderately affected by treatment and the lethal tumor size was reached in 57 generations (1 year).

### Double Bind

**Figure 7:**
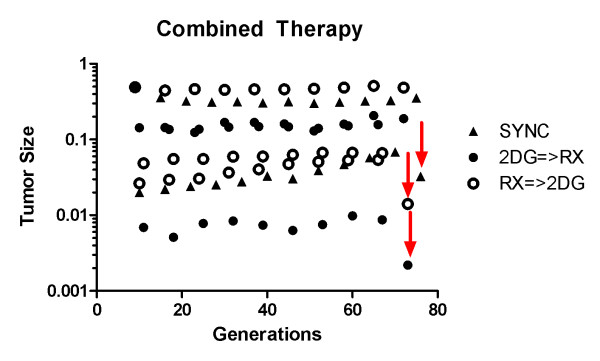
**Comparison of tumor size in different combined therapies**. Comparison of tumor size in different combined therapies. SYNC stands for simultaneous administration of 2DG and Rx, 2DG => Rx corresponds to administration of 2DG followed by Rx, and Rx => 2DG corresponds to administration of Rx followed by 2DG. Red arrows represent final high Rx dose of MTD in one unique dose in order to try tumor eradication.

**Figure 8:**
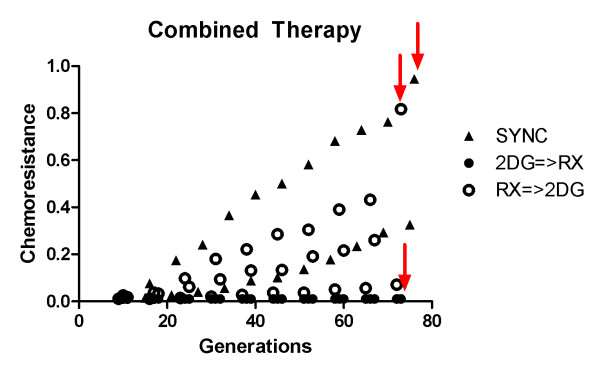
**Comparison of tumor average chemoresistance in different combined therapies**. Comparison of tumor average chemoresistance in different combined therapies. Chemoresistance steadily increases in simultaneous administration, but still more slowly than MTD. After final high dose bolus only 2DG => Rx strategy's tumor remains sensitive. Legend keys are SYNC for simultaneous administration of 2DG and Rx, 2DG => Rx for administration of 2DG followed by Rx, and Rx => 2DG corresponds to administration if Rx followed by 2DG. Red arrows represent final high Rx dose of MTD in one unique dose in order to try tumor eradication.

For all three scenarios tested of combined therapy, at the end of 75 generations (~1.5 years) a high intensity dose was applied to try and eradicate the tumor. This bolus is identified in figures 7 and 8 as red arrows and was used to test if the prolonged double bind treatment could eventually eradicate the chemoresistant subpopulation to the point that a high dose treatment could eventually lead to the possibility of cure even in a tumor originally with chemoresistant cells.

#### Synchronized

The simultaneous administration of 2-deoxy-glucose and chemotherapy led to better results than chemotherapy alone, with most of dead cells being the ones in the outer rim of tumor, and thus environmentally sensitive. The lethal size was reached after 210 generations (4 years), an increase in 15% of life span with reduced chemotoxicity thanks to lower bolus density (20% of MTD).

The overall size of tumor is kept stable for longer (figure 7), in contrast with MTD which drastically reduces tumor size initially but then allows its regrowth. Unfortunately, this treatment also causes rise of chemoresistance (figure 8) that will cause tumor to cease to respond to therapy.

**Figure 9:**
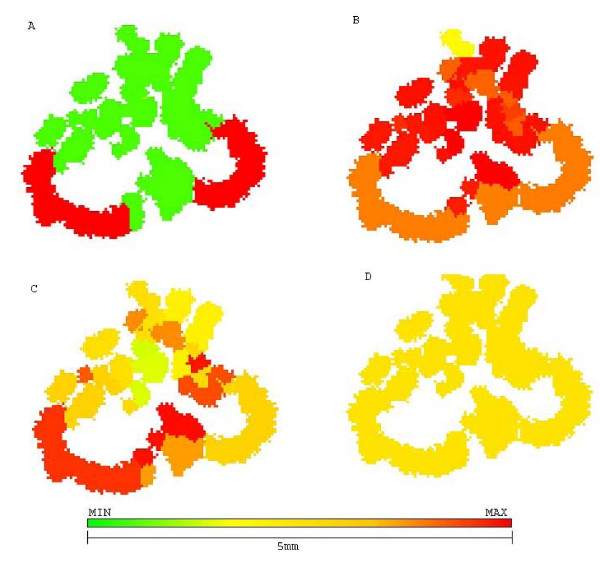
**Morphology and phenotypic distribution in tumor treated with chemotherapy in tandem with glucose competitor at step 75**. Morphology and phenotypic distribution in tumor treated with chemotherapy in tandem with glucose competitor at step 75. A represents chemoresistance (red resistant and green sensitive), B represents hyperglycolysis (red high and green low glycolysis), C shows acid resistance (red for resistance to low pHe and green for sensitivity) and D shows quiescent cells (green) and proliferative (yellow). The chemosensitive population is not capable of completely engulfing resistant population what allows faster remission.

The morphology of a tumor treated with synchronized therapy (figure 9) shows how the chemoresistant population is able to break free from tumor center but still has to compete with a population of sensitive cells.

#### Chemotherapy followed by 2DG

Chemotherapy followed by glucose competitor leads to better results than the simultaneous administration, with smaller tumor size during treatment (figure 7), lower chemoresistance (figure 8) and a life span of 600 generations (~11 years) representing and improvement of 3-fold as compared to MTD.

**Figure 10:**
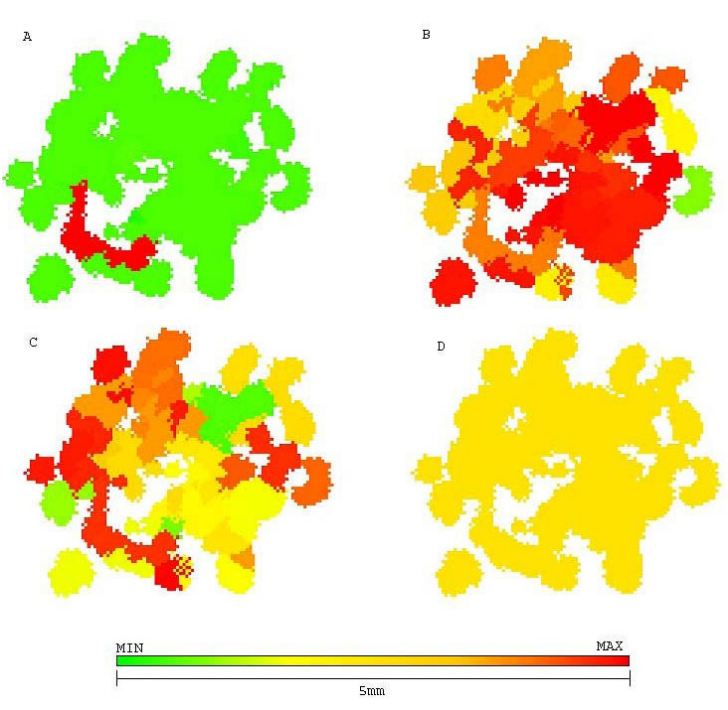
**Morphology and phenotypic distribution in tumor treated with chemotherapy followed by glucose competitor at step 72**. Morphology and phenotypic distribution in tumor treated with chemotherapy followed by glucose competitor at step 72. A represents chemoresistance (red for resistant and green for sensitive), B represents hyperglycolysis (red for high and green for low glycolysis), C shows acid resistance (red for resistance to low pHe and green for sensitivity) and D shows quiescent cells (green) and proliferative (yellow). The extra generation without chemotherapy allows regrowth of sensitive population enough to encompass and delay growth of resistant cells.

The better performance of this approach can be explained by the morphology of figure 10 showing how sensitive cells are kept at a higher proportion if compared to resistant cells. This sensitive population is thus more capable of surrounding resistant cells and slow down their growth.

**Figure 11:**
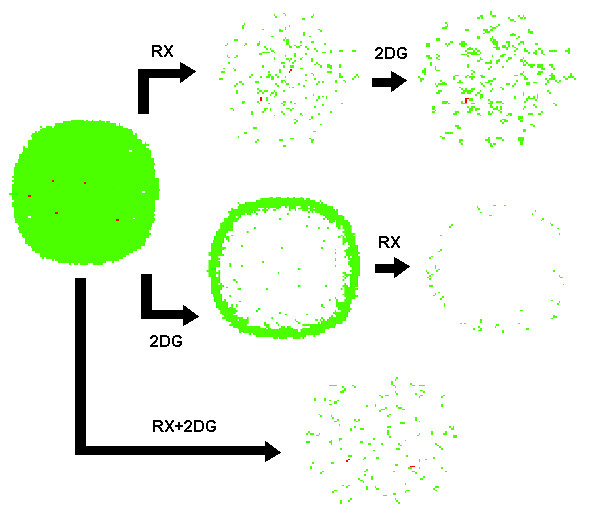
**Three different ways of combining chemotherapy and 2-deoxy-glucose**. Three different ways of combining chemotherapy and 2-deoxy-glucose: (a) First chemotherapy and later 2DG, (b) 2DG first followed by chemotherapy and (c) both administered at the same time. The first approach kills most of the sensitive cells of tumor but misses some of the resistant ones, as a consequence, the tumor perfusion is improved and more oxygen and glucose is available to the remaining cells, neutralizing the effect of 2DG. The simultaneous administration of Rx and 2DG leads to the same scenario, with sensitive and resistant cells left in a more oxygenated porous tumor mass. In both scenarios, with resistant cells free from the tumor core where they were trapped, the fraction of resistant cells will grow and tumor will slowly become more resistant. The strategy that is rewarded with most benefits is the administration of glucose competitor followed by chemotherapy, what produces a smaller sensitive tumor.

Figure 11 shows the comparison of these two combined treatments and how in the case of synchronized therapy, resistant cells are left with more space to regrow while in the Rx => 2DG the extra generation between two chemotherapeutic sessions allows sensitive cells to regrow and trap resistant ones.

#### 2DG followed by chemotherapy

The use of 2-deoxy-glucose followed by chemotherapy presented the most promising results. While reducing chemoresistance (figure 8), tumor size was kept stable (figure 7) and at the end of simulations, at generation 72, no more resistant cells were present.

Figure 11 shows a sequence of steps of original tumor being treated first with 2-deoxy-glucose and next with chemotherapy. This sequence describes how this approach is different from previous ones, as the cells in center of tumor are first led to energy depletion and then chemotherapy acts on killing the ones in outer regions.

**Figure 12:**
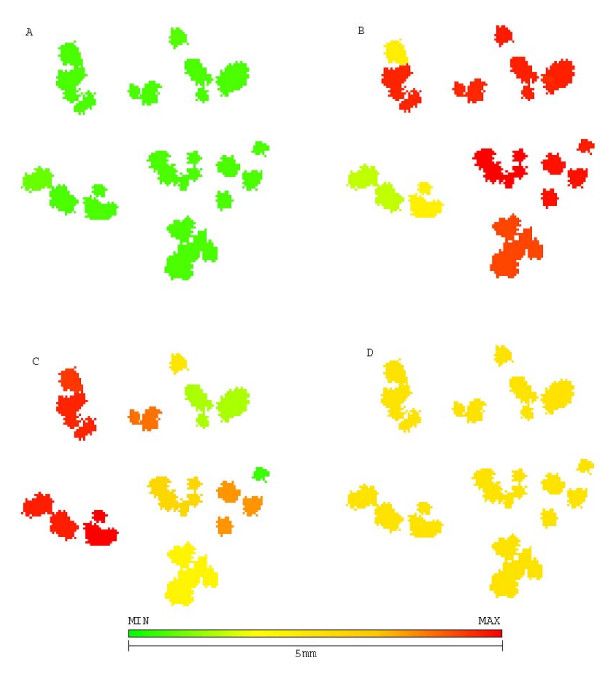
**Morphology and phenotypic distribution of tumor treated with 2DG followed by chemotherapy at step 72**. Morphology and phenotypic distribution of tumor treated with 2DG followed by chemotherapy at step 72. A represents chemoresistance (red resistant and green sensitive), B hyperglycolysis (red high glycolysis and green low), C shows acid resistance (red resistance to low pHe and green sensitivity) and D shows quiescent cells (green) and proliferative (yellow). The "pulverized" morphology grants a higher surface-to-volume ratio what increases efficiency of chemotherapy. All resistant cells have been removed during treatment by energy depletion.

The morphology of this tumor (figure 12) also shows how this approach pulverizes the tumor in smaller volumes with higher surface-to-volume ratio, increasing efficiency of chemotherapy. This pulverized morphology is result of 2-deoxy-glucose energy depletion, preventing tumor cells from existing in hypoxic environments.

### Final Bolus

To asses if tumors can be eradicated after 1.5 years of treatment, a high dose bolus of MTD was used in three cases of combined therapy. The three tumors responded with decrease in volume: 0.35 to 0.03 for synchronized, 0.49 to 0.01 for chemotherapy followed by 2DG (Rx => 2DG) and 0.19 to 0.002 for 2DG followed by chemotherapy (2DG => Rx).

The tumor was virtually eradicated for the last strategy with 22 cells left, all sensitive to therapy, while chemoresistance was increased in the two others.

## Discussion

This work confirms the prediction of previous mathematical models and simulations [18,38] that suggested that administration of chemotherapy with the goal of tumor stabilization instead of eradication would yield better results (longer subject survival) than the use of maximum tolerated doses.

This strategy assumes that resistant cells are already present in the tumor and will replace the sensitive cells killed during treatment. This scenario is impervious to standard MTD treatment and is the strength of this innovative strategy. However, the current models do not take into account the potential mutagenic effects of the chemotherapy - a process that could increase the evolution of resistance. This will be the subject of future work.

The simulations described in this work demonstrate that chemoresistant cells are eventually trapped inside tumor as faster replicating sensitive cells outgrow them. The sensitive cells then will serve as a shield holding these resistant cells at bay until they are killed by chemotherapy and the resistant cells are again free to proliferate.

If only the minimum necessary number of sensitive cells is killed by chemotherapy, a sufficient number will be available to outgrow the resistant population and keep it trapped inside tumor.

**Figure 13:**
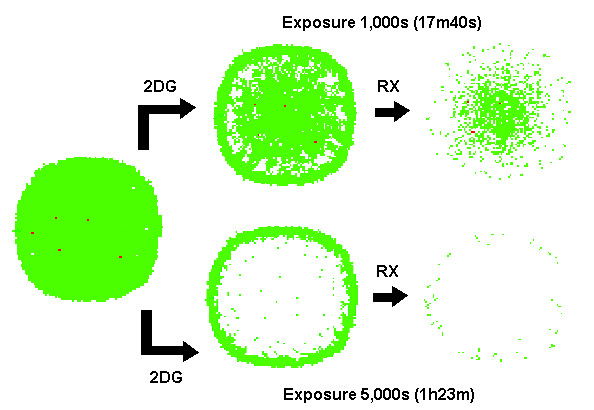
**Drug concentration is not the only factor defining efficiency in tumor cells eradication**. Drug concentration is not the only factor defining efficiency in tumor cells eradication. The diffusion of species from blood vessels to the interior of avascular tumors creates gradients of concentration that favors survival of cells further from tumor-host interface. The exposure of the tumor model to 2DG during 5,000 seconds is enough to produce energy depletion in most cells inside tumor but can only kill a fraction of cells in intermediate region if exposed to only 1,000s. This exposure time is crucial for the efficiency of a drug such as 2DG which acts mainly in inner regions of tumor.

These simulations confirm that the time for reaching steady-state distribution of drug in solid tumors is in the same order as half-life of chemotherapeutics. Figure 13 shows the effect of exposing the same tumor to same bolus intensity of 2-deoxy-glucose (3 mM) and chemotherapy (5 μM) during 1,000 and 5,000 seconds respectively. While shorter exposition was able to kill most of cells in outer rim of tumor, only the longer exposure was capable of affecting the chemoresistant cells, and break up the tumor morphology.

Research with liposomes [30] showed how increasing half-life of drugs in body can significantly increase its efficiency, especially drugs that target the cells within tumor core, such as 2-deoxy-glucose.

Our models also address another major barrier for chemotherapy effectiveness - the reduced sensitivity of quiescent cells in interior of avascular or poorly perfused tumors. Because the drug concentrations vary up to two orders of magnitude [37] between healthy tissue and tumor center during transients, even the maximum tolerated dose may be too low to kill the tumor cells within this region of microenvirnmental resistance.

The use of 2-deoxy-glucose to target cells in ischemic and hypoxic regions of a tumor was proposed almost three decades ago but to our knowledge, no quantitative analysis had been performed on the effect of 2DG on tumor metabolism and on optimal strategies to exploit synergy between chemotherapy and energy restriction.

Our simulations indicate that the simultaneous administration of chemotherapy and 2-deoxy-glucose, the approach used in prior trials, does not optimize treatment outcome because, as both drugs diffuse through tumor towards its center, the tumor cells on the outer rim start dying due to chemotreatment. As these cells die, their mitochondria shut down and more oxygen and glucose can diffuse towards tumor center, cancelling the effect of 2-deoxy-glucose.

The simulation's results suggest that 2-deoxy-glucose has its maximum effect when the tumor exterior is intact and there is a minimum number of cells between the healthy vascularized tissue and the targeted tumor cells in order to its energy depletion effect to act.

This conclusion inspired us to simulate administration first of the glucose competitor followed by chemotherapy. This strategy showed its benefits when at first the tumor center was replaced by a core of starving dying cells -including many of the chemoresistant cells- and then had its exterior rim pulverized by chemotherapy: the repetition of this treatment eventually led to a tumor with no chemoresistant population and thus potentially curable.

## Conclusions

These results can be generalized into an important conclusion that the optimal therapy must look beyond specificity and immediate cytotoxicity of a chemotherapeutic drug. The strategy for the treatment must include understanding of the evolution and the mechanism for resistance and explicit inclusion of strategies to suppress or exploit these evolutionary dynamics.

This work proposes that most promising therapies should be those that manipulate the evolutionary forces that enforce tumor cell fitness towards making these cells less fit than normal cells, as described in this work.

The robustness of tumors comes in part from its genetic and phenotypic variation but also from its environmental heterogeneity. So far, chemotherapy and radiotherapy specialists have faced this heterogeneity as obstacles to be overcome while we propose to exploit these properties and use environmental barriers to trap the tumor in its own microenvironment and reduce its resistance to treatment.

## Appendix A: Computational Model

The solid tumor model for growth and chemotherapy response was simulated using TSim software http://www.i-genics.com, which is available upon request. This software was developed ad hoc for simulating biological systems with cells interacting in a microenvironment.

This model was represented as a fixed-lattice 3D cellular automaton where each cell has its own set of inheritable phenotypes. All cells are incompressible and represented as cubic volumes with 25 μm of side. Cell motility was also abstracted and the "movement" of the tumor is based solely on cell replication and death.

The simulation is composed of three steps: the first consists in cells dying or replicating at every generation (weeks between each of them); each replication or cell death decision is taken based on extracellular concentration of species and intracellular ATP production rate. The extracellular concentration of species depends on the equilibrium between the production/consumption of these species and the diffusion of these species from blood vessels where they are produced (O_2_, glucose, bicarbonate anions) or consumed (H^+^, CO_2_).

Each simulation consisted in multiple generations, each generation corresponding to one week, enough for the model to reach steady-state between metabolism and diffusion of species between two generations. This steady state was calculated in a transient of 5,000 steps, each one corresponding to 1 second in order to simulate the transient of drug concentration in blood and the diffusion of drug into tissue and tumor.

The diffusion of species was calculated numerically using the heuristic described in [23].

Vascularization was represented as blood vessels forming a cubic mesh at a distance of ~4 mm (150 cells) cells from the tumor's center. This vascularization was used as boundary condition: in these vessels oxygen partial pressure, glucose concentration, pH and bicarbonate concentrations were kept at a fixed value.

Cells can proliferate, die or remain quiescent depending on their ATP production rate or extracellular pH (pHe). Should the pHe value be below 7.1 (for acid sensitive cells) or 6.9 (for acid resistant cells), the tumor cells would become quiescent and would not replicate until the next cell cycle when energy and pHe restrictions are satisfied. Tumor cells would die when exposed to pHe below 6.8 (for acid sensitive) or 6.6 (for acid resistant).

At every generation the probability of a cell replication increases as its ATP production is above a minimum level of 0.85 uM/s [28] and plateaus at 8.5 uM/s, for ATP production rates lower than the minimum value the cell dies due to starvation as described in equation 1:(1)

Each cell possess its own energetic metabolism simplified to aerobic conversion of one molecule of glucose into 36 molecules of ATP and 6 of CO_2 _or anaerobically into two molecules of ATP and 2 of lactic acid. The anaerobic or aerobic paths are chosen depending on the availability of oxygen in extracellular environment according to equations 2 to 7:

Glucose aerobic metabolism in cytoplasm and mitochondria:(2)

Glucose anaerobic metabolism in cytoplasm:(3)

In hypoxic conditions (excess of glucose):(4)

In hyperoxic or hypoglycemic conditions (excess of O_2_):(6)

ATP_ANAER _and ATP_AER _correspond to the production rate of ATP anaerobically and aerobically respectively and their sum is the total ATP production rate.

## Competing interests

The authors declare that they have no competing interests.

## Authors' contributions

RAG participated in the elaboration of the biological question and in the review of the manuscript. AS developed the computer model and wrote the manuscript.

## Reviewer's Comments

### Reviewer #1: Mark P Little

Although much data is referenced by the authors for the various model parameters, there is little biological justification presented for certain crucial modelling assumptions. In particular, fundamental to the model is the assumption that there are two populations of cells, one chemo-sensitive and highly proliferative, the other chemo-resistant and more slowly proliferative. However, little evidence is presented for this crucial assumption. Can the authors provide such evidence?

We thank Dr Little for his remarks and agree that not all forms of chemotherapy will incur in a cost of resistance and thus make resistant cells less proliferative. The hypothetical therapy proposed in this work, however, targets cancer cells that are more proliferative than normal host cells. This definition of target automatically confers resistance to tumor cells that proliferate more slowly. These cells, even in absence of treatment will be minority in the tumor (due to their slow proliferation) and will eventually remain trapped in the interior of the tumoral mass.

This is a first simplification from this model since there are other mechanisms of multi-drug resistance- like efflux pumps, for instance- which may be energetically taxing but not directly affect the proliferation rate in an environment rich in nutrients. The second simplification is that there might be gradients of proliferative and resistant phenotypes, the use of two subpopulations however allowed us to infer the proportion of resistant and sensitive cells directly from the overall tumor resistance (Figures 3 and 8).

### Reviewer #2: Marek Kimmel

*The paper advocates, based on biological observations and mathematical modeling, a new strategy of tumor chemotherapy by combination of agents, which jointly protect the balance between the sensitive and resistant phenotypes among cells constituting a tumor*.

*This concept seems to amount to a type of sophisticated maintenance therapy, the examples of which are not entirely unknown in current oncology. Perhaps the authors might discuss some examples of maintenance therapy in existence, which work in a similar way*.

*Counterexamples will also be productive for elucidation of the unique features of the approach proposed*.

We thank Dr Kimmel for his remarks and suggestions on this work.

There are two basic ideas proposed in this article: (1) the sensitive share of the population of a solid tumor (the vast majority of the tumor mass in cancers that normally respond to therapy at a first moment) could be used to slow down the growth of the resistant mutants and thus delay recurrence, and (2) the use of currently available drugs could be optimized if administered in a way that each drug modifies the tumor microenvironment (tumor + stroma + vessels + growth factors) in order to sensitize it to the next drug.

The first concept was explicitly proposed by Gatenby et al. (2009) [39] and labelled "Adaptive Therapy", which advocates the use of the minimum amount of drug required to keep the total tumor size stable. Computer simulations and animal models were used to test this concept, which was extended in this work where the simulations include the spatial distribution of these cells missing in our previous work.

The second concept is slightly different from what is currently done in clinical trials where different drug combinations are tested in order to find protocols that could delay recurrence in incurable diseases. In Multiple Myeloma, for instance, there is a school of thought which proposes to use the maximum number (and amount) of drugs available in the beginning of the treatment, while a second opposite approach dictates to save drugs for later and thus avoid tumors that recur resistant to all currently available drugs [40]. What is missing in this discussion, however, are studies showing exactly how the disease progresses after treatment with each drug, how the subpopulations of the primary tumor, metastases and circulating tumor cells are selected and how each of these microenvironments are changed.

Such studies, however, require realistic quantitative models of both disease progression and in vivo response to therapy. Building such models is a great challenge since most quantitative data (dose response curves) are obtained in vitro using cell lines, which cannot fully represent the phenotypic and environmental heterogeneity of real tumors.

What we show in this work is only a proof of concept of how much more powerful therapies could become, if only we knew exactly how the treatment is affecting the microenvironment and in which direction its is being driven (towards cure, recurrence or maintenance).
